# Effectiveness of a harmonica-integrated, tele-supervised home-based pulmonary rehabilitation program on lung function and comprehensive health outcomes in patients with chronic obstructive pulmonary disease: a randomized controlled trial protocol

**DOI:** 10.3389/fpubh.2025.1541866

**Published:** 2025-01-29

**Authors:** Qiuxuan Zeng, Xiaohong Lin, Wenli Chen, Daniel Yee Tak Fong, Junxin Li, Jiaying Li

**Affiliations:** ^1^National Center for Respiratory Medicine, Guangzhou Institute of Respiratory Health, The First Affiliated Hospital of Guangzhou Medical University, Guangzhou, China; ^2^School of Nursing, University of Hong Kong, Hong Kong, Hong Kong SAR, China; ^3^School of Nursing, Johns Hopkins University, Baltimore, MD, United States

**Keywords:** chronic obstructive pulmonary disease, harmonica playing, music therapy, pulmonary rehabilitation, tele-supervision, home-based training

## Abstract

**Introduction:**

Harmonica playing mimics pursed-lip breathing and strengthens respiratory muscles. Combined with music therapy, it may improve both pulmonary and mental health in chronic obstructive pulmonary disease (COPD) patients, though its effects are not well understood. This protocol outlines a randomized controlled trial (RCT) to evaluate the effectiveness of integrating harmonica playing into pulmonary rehabilitation (PR) programs.

**Methods and analysis:**

This single-center, two-arm RCT will be conducted at a tertiary hospital in Guangzhou, China. A total of 248 adult patients (with a clinical diagnosis of COPD but without severe comorbidities, significant cognitive impairments, and prior experience with the intervention components) will be randomized in a 1:1 ratio to either a harmonica-integrated PR group (intervention) or a standard PR group (control) for 6 months of home-based, tele-supervised training. The intervention will incorporate harmonica sessions in addition to standard PR exercises (breathing and physical exercises). Both groups will undergo in-hospital training sessions, supplemented by daily home practice under remote supervision by PR staff. The primary outcome is lung function (measured by FEV_1_%), while secondary outcomes include respiratory muscle strength, exercise capacity, fatigue, dyspnea, symptom burden, mental health, self-efficacy, quality of life, social support, adherence, and patient satisfaction. Statistical analyses will employ mixed-effects models with an intention-to-treat approach.

**Conclusion:**

This trial will evaluate the efficacy of a harmonica-integrated, home-based PR program with tele-supervision for COPD patients on lung function, respiratory muscle strength, exercise capacity, and overall health. If effective, it could offer a novel, affordable, and accessible home-based PR approach for COPD management.

**Trial registration number:**

ClinicalTrials.gov: NCT05995847.

## 1 Introduction

Chronic obstructive pulmonary disease (COPD), the third leading cause of death globally, is characterized by dyspnea, cough, and sputum production, often accompanied by peripheral muscle dysfunction, cardiovascular disease, anxiety, depression, and chronic fatigue (1, 2). Pulmonary rehabilitation (PR), a cornerstone of non-pharmacological COPD treatment, typically integrates self-management education, respiratory training, psychosocial support, and structured exercise, effectively improving lung function, alleviating symptoms, and enhancing quality of life (3).

Respiratory training is essential in PR, strengthening respiratory muscles and alleviating dyspnea (4). However, certain traditional exercises (e.g., Positive Expiratory Pressure Therapy, Threshold Inspiratory/Expiratory Muscle Training) can be monotonous and difficult to sustain over time, particularly due to their repetitive, non-interactive nature (5, 6). Harmonica playing provides a novel alternative by combining pursed-lip and diaphragmatic breathing with controlled airflow, simulating respiratory training through an engaging, interactive musical activity that reduces monotony and enhances motivation (7, 8). Beyond physiological benefits, music induces positive emotions, reduces stress, and fosters a sense of accomplishment (9). Furthermore, harmonicas are cost-effective (10), with a 24-hole tremolo model from a reputable Chinese brand priced around USD 4.30. This is much cheaper than respiratory training devices like the XEEK Kuner (USD 430) and Breath Home (USD 428). Portable and requiring no extra equipment or space (11), the harmonica further minimizes costs, making it a financially advantageous adjunct to PR.

However, the benefits of harmonica playing in improving health outcomes for COPD patients remain incompletely explored. Three preliminary studies have suggested potential improvements in respiratory muscle strength, exercise capacity, breathing control, and quality of life (12–14). Nevertheless, these studies suffer from significant methodological limitations, including lack of randomization (12), small sample sizes (from 9 to 13) (12–14), and inconsistent practice regimens (10–40 min daily) without standardized guidelines on home practice duration or frequency (12–14). The studies had inadequate follow-up periods (13) and no systematic post-intervention monitoring in others (12, 14), thereby limiting the assessment of long-term impacts. Moreover, they mainly assessed physiological outcomes, like spirometry, inspiratory and expiratory muscle strength, and breathlessness, ignoring functional measures like the six-minute walk distance (6MWD) (15) and inadequately addressing psychological and social factors such as adherence and wellbeing (12–14). These gaps underscores the need for a robust randomized controlled trial with comprehensive outcome measures to evaluate harmonica-integrated PR for COPD.

This study utilizes a rigorous randomized controlled trial (RCT) design with a large sample size, extended intervention period, standardized training protocol, and comprehensive outcomes, addressing the limitations of previous research. It aims to provide robust evidence on the physiological, functional, and psychological benefits of a harmonica-integrated, tele-supervised, home-based PR program for COPD patients. The primary outcome is lung function, with secondary outcomes including respiratory muscle strength, exercise capacity, fatigue, dyspnea, mental health, self-efficacy, quality of life, adherence, and satisfaction—addressing both physiological and psychosocial factors. Findings may inform global COPD management guidelines, positioning harmonica-integrated PR as a cost-effective, accessible, and engaging alternative, particularly in areas with limited access to traditional rehabilitation services.

## 2 Methods and analysis

### 2.1 Study aims

The primary objective of this study is to assess the effectiveness of a harmonica-integrated, tele-supervised, home-based PR program on lung function in patients with COPD, compared to a standard PR program without harmonica integration. Secondary objectives include evaluating the intervention's effects on respiratory muscle strength, exercise capacity, fatigue, dyspnea, symptom burden, mental health, self-efficacy, quality of life, social support, adherence, and satisfaction.

### 2.2 Study design

**Figure 1:**
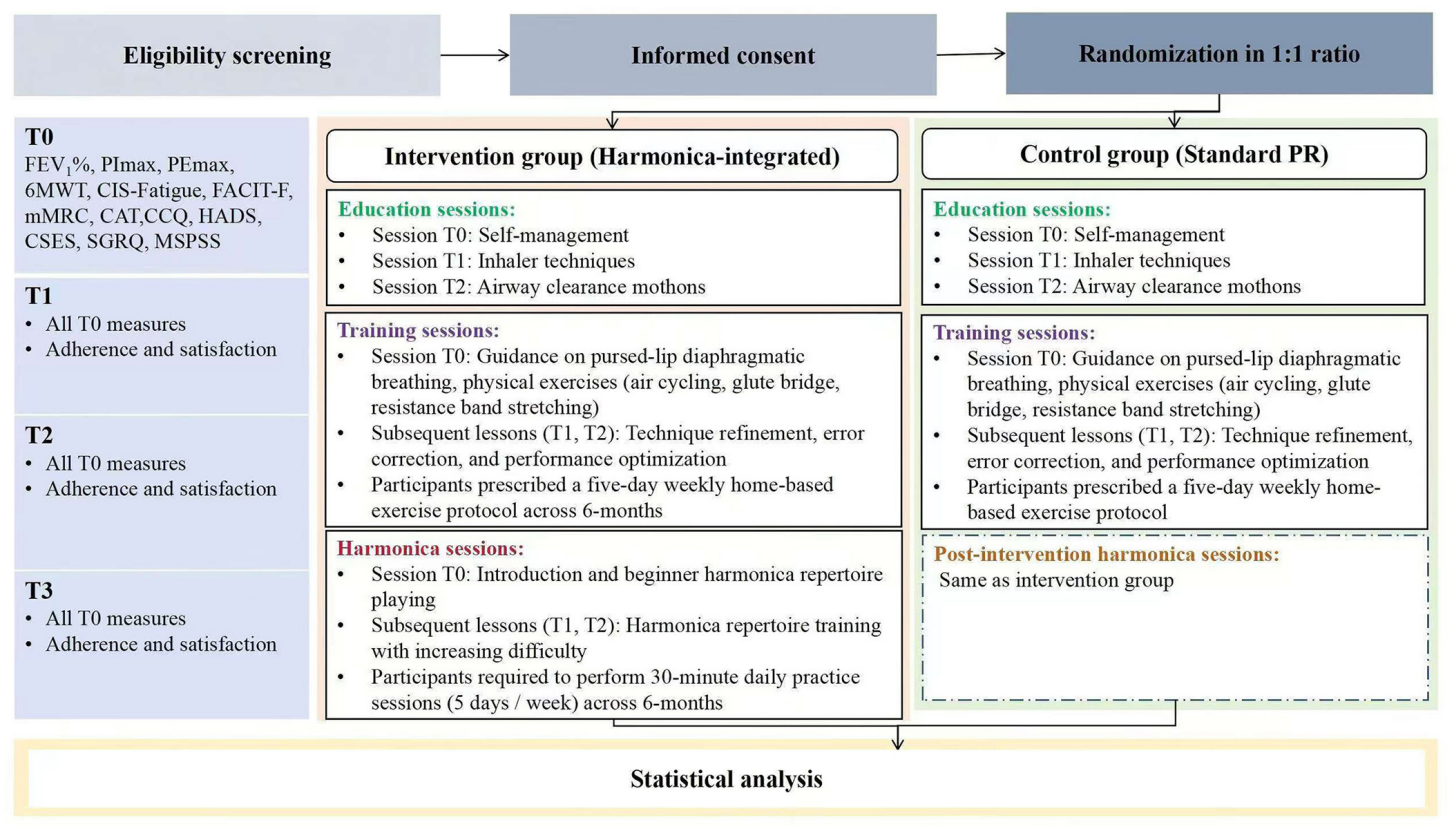
Flowchart of the randomized controlled trial protocol. FEV_1_%, Forced expiratory volume in one second of predicted; PImax, maximum inspiratory pressure; PEmax, maximum expiratory pressure; 6MWT, Six-Minute Walking Test; CIS-Fatigue, Checklist Individual Strength-Fatigue; FACIT-F, Functional Assessment of Chronic Illness Therapy-Fatigue; mMRC, modified Medical Research Council; CAT, Chronic Obstructive Pulmonary Disease Assessment Test; CCQ, Clinical Chronice Obstructive Pulmonary Disease Questionnaire; HADS, Hospital Anxiety and Depression Scale; CSES, Chronic Obstructive Pulmonary Disease Self-Efficacy Scal; SGRQ, St. George's Respiratory Questionnaire; MSPSS, Multidimensional Scale of Perceived Social Support; T0, baseline; T1, 1 month post-baseline; T2, 3 months post-baseline; T3, 6 months post-baseline.

A single-center, two-arm study will be conducted at a tertiary hospital in Guangzhou, China to compare the effects of a harmonica-integrated, tele-supervised home-based PR program with those of a standard PR program on lung function and overall health in COPD patients. A total of 248 adult participants, clinically diagnosed with COPD but free of severe comorbidities, significant cognitive impairments, and prior exposure to the intervention components will be enrolled. The intervention will span 6 months, with clinical assessments performed at baseline (T0, the day the intervention begins), and 1 month (T1), 3 months (T2), and 6 months (T3) post-baseline. Figure 1 illustrates the participant flow through the study. This study registered at ClinicalTrials.gov (NCT05995847).

### 2.3 Eligibility and sample size

Eligible patients must have a clinical diagnosis of COPD and a post-bronchodilator forced expiratory volume in one second (FEV_1_) to forced vital capacity (FVC) ratio of < 70%. Exclusion criteria include recent COPD exacerbations (within 6 weeks), resting oxygen saturation (SpO_2_) below 88% on room air or inadequate oxygenation despite supplementation; as well as severe dyspnea with Modified Medical Research Council (mMRC) grade 4, recent myocardial infarction, unstable angina, severe arrhythmias (e.g., ventricular arrhythmias or rapid atrial fibrillation), or pulmonary hypertension with right heart failure. Additional exclusions include comorbidities affecting physical activity (e.g., neurological disorders, cancer, significant musculoskeletal issues), cognitive impairments, and inability to communicate in Chinese. Furthermore, those with prior participation in PR within 6 months, or regular experience playing harmonica or similar wind instruments, are excluded to ensure baseline equivalence, minimize learning and behavioral biases, and avoid psychological effects, thereby enhancing the validity and reliability of the study outcomes.

Based on an anticipated minimum clinically important difference (MCID) of a 4% increase in percent predicted FEV_1_ (FEV_1_%) and a standard deviation of 10% (16), each group will require 99 participants to achieve 80% power at a 0.05 two-tailed significance level. Considering a 20% dropout rate, the study plans to enroll 248 patients (124 per group) (17).

### 2.4 Recruitment

Participants will be recruited using a comprehensive, multifaceted approach to ensure a broad and inclusive reach. Recruitment efforts will include advertisements in the hospital's outpatient clinics, referrals from healthcare providers, and telephone outreach to former outpatient and inpatient COPD patients. In addition, we will employ online recruitment strategies, such as WeChat and community outreach. The research team will meticulously screen interested individuals based on stringent inclusion and exclusion criteria to determine their eligibility. Enrollment will occur on a rolling basis, allowing eligible candidates to join the study as they meet the criteria and space is available. All eligible candidates will receive detailed information about the study through an information letter and an informative flier. Those who agree to participate will provide written informed consent as part of the enrollment process.

### 2.5 Randomization

Participants will be randomly assigned in a 1:1 ratio to either the intervention or control group using a computer-generated randomization sequence developed by an independent statistician unaffiliated with the recruitment process. To ensure allocation concealment, the sequence will be sealed in opaque, sequentially numbered envelopes. Upon enrollment, the recruitment coordinator, who does not have prior access to the allocation sequence, will open the corresponding sealed envelope to assign each participant to their respective group. The recruitment coordinator will then inform participants of their group allocation and provide the necessary materials, including disease education manuals, rehabilitation training diaries, elastic bands, and portable pulse oximeters for monitoring oxygen saturation during exercises. Participants allocated to the harmonica-integrated group will additionally receive a harmonica. Additionally, the recruitment coordinator will communicate the time and location of each session to the participants.

### 2.6 Blinding

This study employs a partially blinded design to minimize potential biases. Care providers responsible for delivering the interventions—including rehabilitation instructors, harmonica instructors, home-training supervisors, and home-training recorders—are blinded to group allocations and the overall study protocol. They are informed only of the specific treatments they are to administer without knowledge of the study protocol and participants' group assignments. Outcome assessors conducting evaluations are blinded to the group allocations to ensure objective assessment of study outcomes. The data analyst performing the final data analysis is also blinded to group assignments to maintain objectivity in data interpretation. Although the recruitment coordinators are aware of group assignments for administrative purposes, they do not engage in any intervention, assessment, or follow-up tasks to minimize potential biases. Due to the nature of the intervention, patients cannot be blinded. However, potential biases will be minimized by standardizing intervention protocols and using objective outcomes as primary measures.

### 2.7 Care for intervention group

The intervention group will undergo a six-month program including education sessions, training sessions, and harmonica sessions. Training and harmonica sessions will be conducted in person at the hospital, followed by daily home-based exercises with continuous tele-supervision and feedback delivered via WeChat. Details are listed in Table 1.

**Table 1:** Components of the care for the intervention and control groups.

**Components**	**Contents**	**In-hospital/home-training**	**Time point**	**Intervention group**	**Control group**
**Education sessions**					
Self-management strategies	COPD self-management knowledge: 1. The pathophysiology, symptoms, progression, and risk factors of COPD 2. Recognizing and avoiding triggers, the importance of regular physical activity 3. Adhering to prescribed medication regimens, using oxygen therapy correctly 4. Lifestyle modifications: smoking cessation, healthy diet, and stress management	In-hospital	T0	√	√
Inhaler techniques	Correct usage protocols for pressurized metered-dose, dry powder inhaler, soft mist inhaler. Daily storage and cleaning of inhalation devices, and measures to prevent adverse reactions	In-hospital	T1	√	√
Airway clearance methods	Airway clearance techniques: 1. The ACBT techniques 2. Postural drainage 3. The OPEP techniques	In-hospital	T2	√	√
**Training**					
Pursed-lip and diaphragmatic breathing	Steps for pursed-lip abdominal breathing: 1. Positioning: Adopt a comfortable position, whether sitting, standing, or lying down 2. Hand Placement: Place one hand on the abdomen and the other on the chest to help monitor the movement of the diaphragm and chest respectively 3. Inhalation: Close the mouth and inhale through the nose. During inhalation, allow the abdomen to expand gently. The hand placed on the abdomen should move outward, while the hand on the chest should remain relatively stationary or move minimally 4. Exhalation: • Purse the lips as if whistling or making an “s-s-s” sound • Exhale slowly through the pursed lips, allowing the abdomen to contract • The hand on the abdomen should move inward during exhalation • The exhalation should be twice as long as the inhalation to ensure effective lung emptying Frequency: 30 times/set, 15 min/day, 5 days/week	In-hospital and home-training	T0 to T3	√	√
Air cycling exercise	Steps for air cycling exercise: 1. Lie supine on the bed with the lumbar and dorsal regions closely adhering to the mattress, placing the hands on both sides of the body 2. Adjust the breathing, then lift both legs and slowly perform a pedaling motion as if cycling 3. Maintain natural respiration throughout the training process and avoid breath-holding. Allow for intermittent rest periods of 10–30 s as needed Frequency: 30 cycles (per leg)/set, 3 sets/day, 5 days/week	In-hospital and home-training	T0 to T3	√	√
Hip bridge exercise	Steps for hip bridge exercise: 1. Lie supine on the bed with the hands placed on both sides of the body 2. Bend the knees and firmly plant the feet on the bed surface, flexing the knee joints to a 90-degree angle 3. Adjust the breathing. During exhalation, contract the abdominal muscles and simultaneously use the gluteal muscles to lift the hips upward, aligning the knees, hips, and shoulders into a straight line 4. Maintain this position for 5–10 s with the abdomen and glutes tightened, while continuing to breathe naturally and avoiding breath-hold in 5. Upon exhaling again, lower the hips back to the bed surface Frequency: 10 repetitions/set, 3 sets/day, 5 days/week	In-hospital and home-training	T0 to T3	√	√
Seated stretching	Steps for seated stretching: 1. Sit upright on a chair with a resistance band looped around a fixed post 2. Grasp each end of the resistance band with the hands, wrapping it around the palms 1 to 2 times. Keep the elbows close to the sides of the body 3. After adjusting the breathing, exhale while pulling the resistance band backward to its maximum range of motion 4. Hold the position briefly, then slowly return to the starting position Frequency: Each movement 10 times/set, 3 sets/day, 5 days/week	In-hospital and home-training	T0 to T3	√	√
Standing stretching	Steps for seated stretching: 1. Stand with feet and hands apart, chest lifted and abdomen tucked in 2. Loop a resistance band around the back and grasp it with both hands, wrapping it around the palms 1 to 2 times 3. Stabilize the scapulae, bend the arms, and place the elbows close to the sides of the body 4. During exhalation, use the force from the chest wall to push the resistance band forward, leading with the arms 5. Hold the position briefly, then slowly return to the starting position Frequency: Each movement 10 times/set, 3 sets/day, 5 days/week	In-hospital and home-training	T0 to T3	√	√
Lower limb stretching	Steps for lower limb stretching: 1. Fold the elastic band in half and loop it around the middle of one foot, passing through the arch. Place the other foot firmly on the elastic band, with the legs slightly apart 2. Hold the elastic band with one hand, wrapping it around the palm 1 to 2 times, and use the other hand to steady oneself against a chair back or tabletop to maintain body stability 3. During exhalation, engage the thigh muscle of the leg with the elastic band to lift the knee upward, resisting the band's tension and raising it as high as possible. As inhalation occurs, slowly lower the leg 4. Switch legs and repeat the above movements Frequency: Each movement 10 times/set, 3 sets/day, 5 days/week	In-hospital and home-training	T0 to T3	√	√
**Harmonica sessions**					
Instrument anatomy	Covers harmonica components—comb, reed plates, mouthpiece—and note arrangement	In-hospital	T0	√	
Maintenance and cleaning	1. Clean regularly: Use warm water and a mild soap to gently wash the harmonica. Avoid using harsh chemicals or hot water 2. Dry thoroughly: Make sure all parts are completely dry before reassembling to prevent reeds from sticking 3. Store properly: Keep the harmonica in a dry, cool place to maintain its condition	In-hospital	T0	√	
Harmonica-based breathing techniques	1. Inhale slowly and deeply using the diaphragm 2. Exhale in a controlled manner, similar to pursed-lip breathing, ensuring steady airflow and clear sound	In-hospital	T0	√	
Hands-on harmonica repertoire teaching	Instructors will select songs of gradually increasing difficulty, providing hands-on teaching for each song and offering personalized guidance. Frequency (home-training): 30 min (no-break)/day, 5 days/week	In-hospital and home-training	T0 to T3	√	

COPD, Chronic Obstructive Pulmonary Disease; ACBT, Active Cycle of Breathing Technique; OPEP, Oscillating Positive Expiratory Pressure; T0, baseline; T1, 1 month post-baseline; T2, 3 months post-baseline; T3, 6 months post-baseline.

#### 2.7.1 Education sessions

The intervention group will participate in three sequential 45-min educational sessions designed to enhance self-management. The initial session (T0) covers disease-specific knowledge and rehabilitation strategies. The second session (T1) focuses on optimal inhaler use, and the final session (T2) introduces advanced airway clearance techniques, building on prior learning. The curriculum progresses incrementally, tailored to participants' learning abilities to gradually improve disease self-management. Additionally, at baseline, each patient will receive disease education manuals for home study.

#### 2.7.2 Training sessions

**Figure 2:**
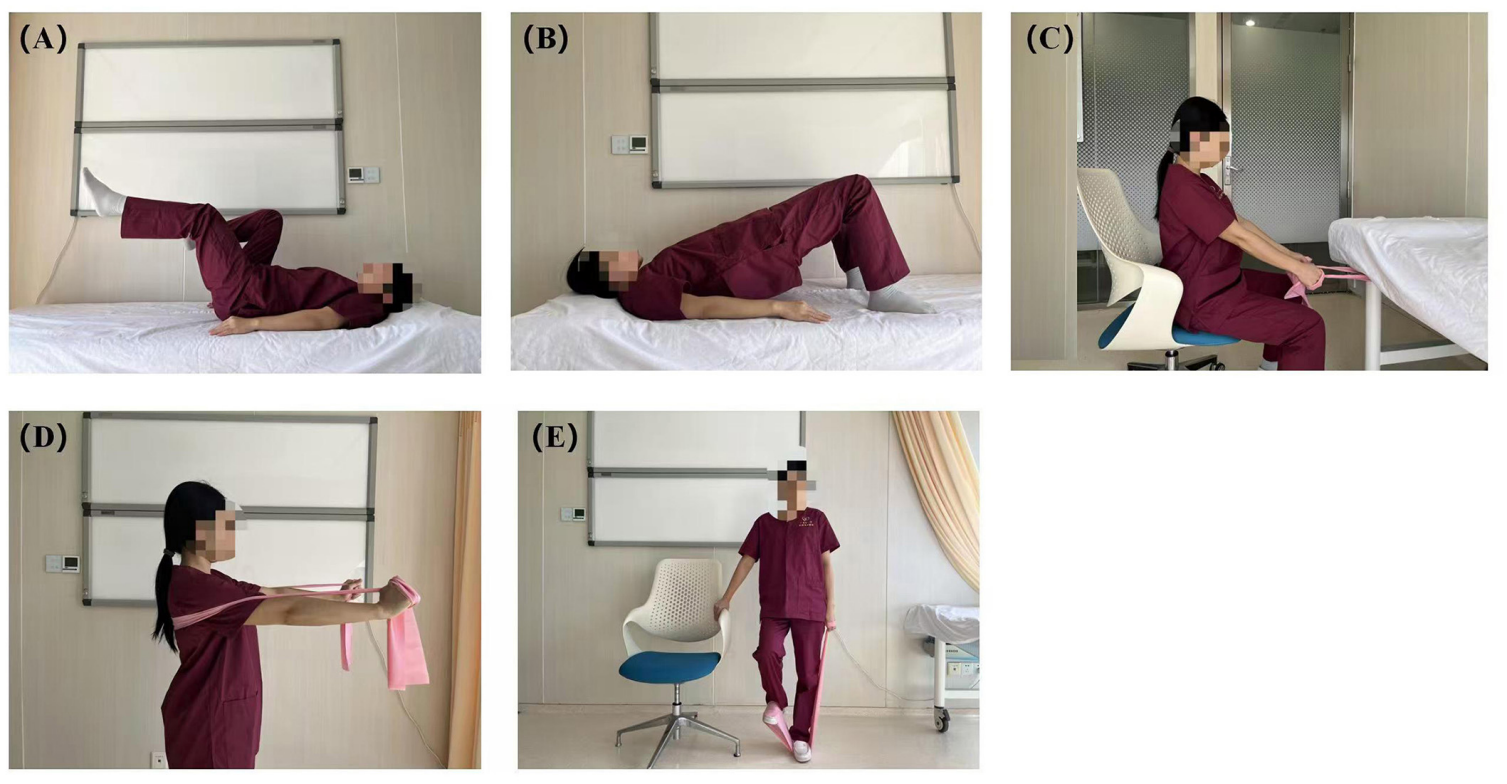
Training exercises included in the program. **(A)** Air cycling exercises; **(B)** hip bridge exercises; **(C)** seated stretching; **(D)** standing stretching; **(E)** lower limb stretching.

The intervention group will participate in three instructor-led training sessions focused on respiratory optimization. The initial session (T0) will provide personalized instruction in breathing techniques (pursed-lip and diaphragmatic breathing) and physical exercises (air cycling exercises, hip bridge exercises, seated stretching, standing stretching, and lower limb stretching), as illustrated in Figure 2. Subsequent sessions (T1 and T2) will concentrate on refining techniques, correcting errors, and optimizing performance. Additionally, participants will adhere to a home-based exercise regimen prescribed for 5 days each week.

#### 2.7.3 Harmonica sessions

Participants in the intervention group will receive three progressive harmonica lessons (T0, T2, T3), conducted by professional music instructors, designed to optimize skill development. The initial lesson (T0) will cover harmonica fundamentals, including instrument anatomy, maintenance and cleaning, harmonica-based breathing techniques, and basic performance skills, establishing a foundation for subsequent lessons. At baseline, simple songs will be selected to build patients' confidence. Participants will be provided with structured written materials outlining practice methods and musical exercises. Subsequent lessons (T2 and T3) will introduce classical songs of increasing difficulty, building on foundational skills and progressively enhancing technical proficiency and enjoyment. Throughout the six-month intervention, participants will engage in a daily 30-min continuous (no-break) practice of a specified set of songs.

#### 2.7.4 Tele-supervision and guidance for intervention group

Upon randomization, a WeChat group will be established for each intervention cohort, including participants themselves, their family members (if they choose), a home-training supervisor and recorder, a rehabilitation instructor, and a harmonica instructor. For participants with limited device familiarity or access, one-on-one personalized WeChat training will be conducted at baseline, including video tutorials, content uploads, and interaction with PR staff. Family members will be trained if needed to assist with remote monitoring.

Participants will upload daily videos of their training and harmonica playing, report training duration, and complete rehabilitation training diaries documenting exercise and harmonica practice times. The supervisor and recorder will independently review the videos to verify adherence, and discrepancies will be resolved through double-checking the video recordings. Additionally, the supervisor and recorder will send daily reminders, track attendance, and note any absences. Rehabilitation and harmonica instructors will conduct real-time video assessments of participants' techniques, providing immediate feedback, personalized guidance, and addressing participant queries.

### 2.8 Care for control group

The control group will participate in a six-month program, which is similar to the intervention group in structure but excludes the harmonica sessions. This group will undergo the same educational and rehabilitation training sessions as the intervention group. The specific activities will be detailed in Table 1.

#### 2.8.1 Supervision and guidance for control group

After randomization, control group participants will be added to a dedicated WeChat group comprising participants, their family members (optional), a home-training recorder and supervisor, and a rehabilitation instructor. The same recording methods and daily prompts will be applied as in the intervention group. The home-training supervisor will review uploaded videos, monitor adherence, send daily reminders, and track training completion. The rehabilitation instructor will conduct real-time video assessments to ensure proper adherence to the training protocols.

### 2.9 Outcomes

Primary and secondary outcomes will be assessed at four key intervals: baseline (T0), 1 month (T1), 3 months (T2), and 6 months (T3) post-baseline, as shown in Table 2. Secondary outcomes related to “adherence and satisfaction” will be measured only at T1, T2, and T3.

**Table 2:** Assessment of primary and secondary outcomes.

**Outcomes**	**Time points**	**Measurement tools and methods**
**Primary outcome**		
Lung function	T0, T1, T2, and T3	Forced expiratory volume in one second percent of predicted (FEV_1_%) 48 h after discontinuing inhaled bronchodilators
**Secondary outcome**		
Inspiratory muscle strength	T0, T1, T2, and T3	Maximum inspiratory pressure (PImax)
Expiratory muscle strength	T0, T1, T2, and T3	Maximum expiratory pressure (PEmax)
Exercise capacity	T0, T1, T2, and T3	6-min Walking Test (6MWT)
Fatigue	T0, T1, T2, and T3	Checklist Individual Strength-Fatigue (CIS-Fatigue) Functional Assessment of Chronic Illness Therapy-Fatigue (FACIT-F) Scales
Breathlessness level	T0, T1, T2, and T3	Modified Medical Research Council (mMRC) Dyspnea Scale
Clinical symptoms	T0, T1, T2, and T3	COPD Assessment Test (CAT)
Clinical control	T0, T1, T2, and T3	COPD Control Questionnaire (CCQ)
Mental health	T0, T1, T2, and T3	Hospital Anxiety and Depression Scale (HADS)
Self-efficacy	T0, T1, T2, and T3	Chronic Obstructive Pulmonary Disease Self-Efficacy Scale (CSES)
Quality of life	T0, T1, T2, and T3	St. George's Respiratory Questionnaire (SGRQ)
Social support status	T0, T1, T2, and T3	Multidimensional Scale of Perceived Social Support (MSPSS)
Adherence and satisfaction	T1, T2, and T3	Recording and self-designed questionnaire

COPD, Chronic Obstructive Pulmonary Disease; T0, baseline; T1, 1 month post-baseline; T2, 3 months post-baseline; T3, 6 months post-baseline.

#### 2.9.1 Primary outcomes

The primary outcome of this study is the change in lung function, measured as FEV_1_% 48 h after discontinuing inhaled bronchodilators. Measurements will be obtained using the portable X1 spirometer (serial no. X1231100300024) from Saikemed (Xiamen) Medical Equipment Co., Ltd. Predicted FEV_1_ values will be calculated based on the “Compilation of Normal Lung Function Values Across China” (Mu Kuijin & Liu Shiwuan, eds.): for males, FEV_1_ = −1.087 – 0.029 × A + 0.033 × H; for females, FEV_1_ = −0.753 – 0.022 × A + 0.026 × H, where A is age (years) and H is height (cm) (18). A clinically significant change is defined as a ≥4% improvement in FEV_1_%, a recognized MCID in COPD research (16). COPD severity will be classified according to the ATS/ERS criteria (2005/2019) based on post-bronchodilator FEV_1_%: mild (≥70%), moderate (60–69%), moderate-to-severe (50–59%), severe (35–49%), and very severe (< 35%) (19).

#### 2.9.2 Secondary outcomes

Secondary outcomes will encompass changes in respiratory muscle strength, exercise capacity, fatigue, breathlessness, clinical symptoms, clinical control, mental health, self-efficacy, quality of life, social support, adherence and satisfaction. Each outcome will be measured using validated tools accordingly to ensure reliable and actionable insights.

##### 2.9.2.1 Inspiratory and expiratory muscle strength

Inspiratory and expiratory muscle strength will be measured as PImax (maximum inspiratory pressure) and PEmax (maximum expiratory pressure) using the X1 spirometer (serial no. X1231100300024).

##### 2.9.2.2 Exercise capacity

The 6-min walk test (6MWT) is a validated measure of exercise capacity in COPD patients, demonstrates reliable performance in the Chinese population and correlates with disease severity and mortality (20). Participants will complete a 6-min walk along a standardized flat corridor, with total distance walked serving as the primary outcome metric. Heart rate and perceived exertion (Borg scale) will be assessed pre- and post-test to evaluate exercise intensity and physiological recovery. Predicted 6MWD (meters) will be calculated using Enright and Sherrill's formulas based on body mass index (BMI) and age: for males, 6MWD = 1,140 – 5.61 × BMI – 6.94 × age; for females, 6MWD = 1,017 - 6.24 × BMI – 5.83 × age. These values will provide a reference for interpreting the actual distance walked (21).

##### 2.9.2.3 Fatigue

Fatigue will be measured using the Chinese version of the Checklist Individual Strength-Fatigue (CIS-Fatigue; Cronbach's alpha of 0.88) and Functional Assessment of Chronic Illness Therapy-Fatigue (FACIT-F; Cronbach's alpha of 0.90) scales. Both scales are validated in Chinese populations and are widely used in assessing fatigue in chronic illness (22, 23). The CIS-Fatigue, a 20-item subscale of the Checklist Individual Strength, employs a 7-point Likert scale with total scores ranging from 8 to 56, where scores < 26, 27–35, and >36 represent normal, mild, and severe fatigue, respectively. The FACIT-F, a 13-item self-reported scale rated on a 5-point Likert scale (“not at all” to “very much”), measures fatigue-related tiredness, weakness, and activity limitations over the preceding seven days. Higher scores on both scales indicate greater fatigue (24).

##### 2.9.2.4 Breathlessness level

The mMRC Dyspnea Scale, with a Cronbach's alpha of 0.82 in China, will assess breathlessness severity on a 0–4 scale. A score of 0 indicates no breathlessness, while 1 represents breathlessness when hurrying or walking uphill. Scores of 2 and 3 correspond to slower walking or stopping for breath after 100 meters or a few minutes on level ground, respectively. A score of 4 indicates breathlessness when dressing or an inability to leave the house. This scale is commonly used in Chinese COPD research and clinical practice, showing strong correlations with pulmonary function and quality of life (25).

##### 2.9.2.5 Clinical symptoms

Clinical symptoms will be evaluated using the Chinese version of the COPD Assessment Test (CAT; Cronbach's alpha of 0.805), a 5-point scale with 8 items assessing cough, sputum, dyspnea, chest tightness, confidence, activity, sleep, and energy. Scores range from 0 to 40: 0–10 (mild), 11–20 (moderate), 21–30 (severe), and 31–40 (very severe) impact. The CAT, validated in the Chinese COPD population, is sensitive to clinical changes and strongly correlates with health-related quality of life (26).

##### 2.9.2.6 Clinical control

Clinical control will be assessed using the Chinese version of the Clinical COPD Questionnaire (CCQ; Cronbach's alpha of 0.90) (27), a 10-item tool evaluating symptoms, function, and mental state. Items are scored on a 7-point scale (0 = no impairment, 6 = most severe impairment), with higher scores indicating poorer disease control. It is validated in Chinese COPD patients and correlates with clinical outcomes and quality of life (28).

##### 2.9.2.7 Mental health

Anxiety and depression will be assessed using the Chinese version of the Hospital Anxiety and Depression Scale (HADS; Cronbach's alpha of 0.78–0.93) (29), a 4-point scale (0–3) tool with 14 items, divided into two subscales: anxiety (HADS-Anxiety 7 items) and depression (HADS-Depression, 7 items). Each subscale is scored from 0 to 21, with categories: 0–7 (normal), 8–10 (mild), 11–14 (moderate), and 15–21 (severe). The HADS is widely validated in Chinese COPD clinical settings as a reliable measure of psychological distress (30).

##### 2.9.2.8 Self-efficacy

Self-efficacy will be measured using the Chinese version of the Chronic Obstructive Pulmonary Disease Self-Efficacy Scale (CSES; Cronbach's alpha > 0.80), which is validated for use in Chinese populations (31). This 20-item, 10-point scale (0–10) assesses symptom management, physical activity, and psychological coping, with total scores ranging from 0 to 200. Higher scores indicate greater self-efficacy in managing COPD (32).

##### 2.9.2.9 Quality of life

The Chinese version of the St. George's Respiratory Questionnaire (SGRQ; Cronbach's alpha of 0.97) will be used to assess subjective quality of life (33). It has been validated for use in COPD patients in China and is an essential tool for capturing the multidimensional impact of COPD on patients' lives, including respiratory symptoms (e.g., cough, sputum, dyspnea), activity limitations (e.g., walking, sports, chores), and disease impacts (e.g., walking, sports, chores). Scores, ranging from 0 (no impairment) to 100 (worst health), are calculated as a weighted average of the individual item scores, with higher scores indicating a greater disease impact (34).

##### 2.9.2.10 Social support

The Chinese version of the Multidimensional Scale of Perceived Social Support (MSPSS; Cronbach's alpha of 0.91) (35) will assess perceived social support from family, friends, and significant others using a 12-item, 7-point Likert scale (1–7). Subscale scores range from 4 to 28, and total scores from 12 to 84, with higher scores indicating greater perceived support. It has shown reliability in the Chinese population and is an effective tool for assessing social support in COPD patients (36).

##### 2.9.2.11 Adherence and satisfaction

Adherence and satisfaction will be assessed at T1, T2, and T3. Adherence will be quantified by recording attendance and exercise completion. Satisfaction will be assessed using a study-specific questionnaire designed to comprehensively capture participants' perceptions.

### 2.10 Statistical analysis

Data will be collected using a dual-entry and verification process, incorporating range checks, logic tests, and source document validation to ensure data integrity (37). All analyses will be conducted with R software version 4.4.0. The primary analysis will follow the intention-to-treat principle (ITT), including all randomized participants in their assigned groups (38). Baseline characteristics will be summarized using descriptive statistics, with continuous variables compared using independent *t*-tests and categorical variables using chi-square tests between the harmonica-integrated PR group and the standard PR care group. Primary and secondary outcomes will be analyzed using mixed-effects models to account for the repeated measures design (39). Sensitivity analyses will include per-protocol analyses focusing on participants who fully adhered to the intervention. Effect sizes and 95% confidence intervals will be reported alongside *p*-values. All statistical tests will be two-tailed, with significance set at *p* < 0.05. Subgroup analyses will be conducted based on pre-specified baseline characteristics as necessary. Cohen's D will be used as the effect size metric, with common thresholds (small: 0.2, medium: 0.5, large: 0.8) (40).

## 3 Discussion

By leveraging the respiratory benefits of harmonica playing—which simulates respiratory training and incorporates the advantages of music therapy—along with the convenience of home-based training and the instant support of tele-supervision, this study aims to evaluate the effectiveness of a harmonica-integrated, tele-supervised home-based PR program. This innovative approach may offer an accessible, affordable, and enjoyable non-pharmacological treatment option for patients with COPD.

With a robust sample size of 248 participants, a six-month intervention, standardized protocols, and comprehensive outcome measures, the study enhances statistical power, generalizability, and long-term assessment (41, 42). Key design features include a partially blinded methodology, where care providers, outcome assessors, and data analysts are unaware of group assignments, minimizing bias and ensuring objective evaluation (43). The home-based approach improves accessibility for remote or mobility-limited patients, reduces travel and costs, and may lower infection risks compared to clinic settings (44, 45). Tele-supervision involves roles such as home-training recorder, supervisor, and instructors, providing real-time guidance and technique correction. Communication via WeChat fosters a supportive community, while video recordings ensure objective adherence data (46). Harmonica training progresses from basic music theory and breathing techniques to advanced playing skills, promoting mastery and enjoyment. Concurrently, comprehensive PR sessions include breathing exercises (e.g., pursed-lip, diaphragmatic) and physical activities (e.g., air cycling, hip bridge, resistance band stretching) to enhance respiratory and overall muscle function (41, 47). Regular technical assessments and introductory guidance support effective home-based training.

This intervention offers scalable advantages over traditional rehabilitation methods by utilizing widely accessible technologies such as smartphones and WeChat, ensuring broad feasibility and seamless integration with existing healthcare systems (48). The harmonica's low cost and portability facilitate implementation in resource-limited settings without the need for specialized equipment. Tele-supervision and remote monitoring enhance efficiency, particularly in areas with limited rehabilitation services (49). To address unfamiliarity with equipment and limited access, one-on-one personalized WeChat training will provided at baseline, including video tutorials, content uploads and interacting with PR staff, with family members trained if needed to assist in remote monitoring.

Several limitations and challenges need attention and efforts to mitigate. The lack of participant blinding may introduce bias, especially for subjective outcomes. Emphasizing objective measures (e.g., lung function tests), blinded data analysts, strict quality control, and adherence to standardized protocols can partially mitigate this. Nonetheless, self-reported outcomes (e.g., satisfaction, quality of life, mental health) remain susceptible to expectation bias, requiring cautious interpretation of related outcomes. Self-selection bias, due to voluntary participation and the novelty of harmonica use, may also affect findings. Recording baseline characteristics and recruiting a diverse participant pool may mitigate this. While video evidence can reduce adherence bias, misreporting may still occur, enhanced supervision and daily confirmations could address this. The six-month duration may increase dropout rates, particularly among severe COPD patients. Regular follow-ups and motivational support may help, but additional recruitment may be necessary if dropout rates jeopardize statistical power. Furthermore, the single-center approach may limit generalizability, necessitating multi-center trials for broader applicability in the future. Failure to stratify participants by Global Initiative for Chronic Obstructive Lung Disease (GOLD) stage may lead to underrepresentation of certain stages, particularly GOLD 4, and potentially skew subgroup analyses. As treatment responses vary by stage, this lack of stratification could confound efficacy assessment. Given COPD's patient heterogeneity, not stratifying may result in inaccurate evaluations of intervention effectiveness among patients at different stages, affecting overall treatment judgment. Future study designs or RCTs should account for this. The absence of long-term follow-up will restrict the assessment of enduring effects, extended follow-up should be included in future trials. While time equivalence between the intervention and control groups is challenging, the primary activities (breathing and exercise training) ensure general time equivalence. Future analyses should consider additional time demands on the intervention group. While volume, frequency, and density are well-defined in our intervention, objective measures of intensity (e.g., breath pressure, mouth/tongue effort) remain unquantified. Inter-participant variability in technique may therefore introduce differences in actual training load, potentially impacting the findings and warranting cautious interpretation. Future research may explore device-based measures (e.g., breath pressure sensors, digital harmonicas) to better capture and standardize the intensity component of this intervention. Moreover the optimal duration and frequency for clinical outcomes are not yet established and should be explored in future trials.

## 4 Conclusion

This study aims to enhance COPD rehabilitation by integrating harmonica playing into a home-based, tele-supervised program. By addressing existing research gaps, it seeks to provide robust empirical evidence for a novel, cost-effective, and accessible intervention strategy for COPD management. The findings may promote music-based therapies in chronic disease management and encourage multidisciplinary approaches that combine respiratory therapy with psychological and social support, thereby expanding and improving COPD rehabilitation strategies and patient outcomes.

## References

[1] Christenson SA Smith BM Bafadhel M Putcha N. Chronic obstructive pulmonary disease. Lancet. (2022) 399:2227–42. 10.1016/S0140-6736(22)00470-6 35533707

[2] Petousi N Pavord ID Couillard S. The Lancet COPD Commission: broader questions remain. Lancet. (2023) 401:1569–70. 10.1016/S0140-6736(23)00556-1 37179114

[3] Schneeberger T Abdullayev G Koczulla AR. Pneumologische rehabilitation. Rehabilitation. (2023) 62:232–47. 10.1055/a-2043-6767 37579755

[4] Rochester CL Spruit MA Holland AE. Pulmonary Rehabilitation in 2021. JAMA. (2021) 326:969–70. 10.1001/jama.2021.6560 34519811

[5] Shi L Liu F Liu Y Wang R Zhang J Zhao Z Zhao J. Biofeedback respiratory rehabilitation training system based on virtual reality technology. Sensors. (2023) 23:9025. 10.3390/s23229025 38005413 PMC10674163

[6] Daynes E Houchen-Wolloff L Barradell AC Greening NJ Singh SJ. The training to improve dyspnoea study- patient experiences of using a high frequency airway oscillating device. Int J Chron Obstruct Pulmon Dis. (2024) 19:1345–55. 10.2147/COPD.S443186 38887676 PMC11182029

[7] Lewis A Conway J Middleton J Startup CK Wyatt J. Playing the harmonica with chronic obstructive pulmonary disease. A qualitative study. Chron Respir Dis. (2022) 19:14799731221083315. 10.1177/14799731221083315 35412384 PMC9008858

[8] de Witte M Pinho ADS Stams GJ Moonen X Bos AER van Hooren S. Music therapy for stress reduction: a systematic review and meta-analysis. Health Psychol Rev. (2022) 16:134–59. 10.1080/17437199.2020.1846580 33176590

[9] Kaasgaard M Rasmussen DB Andreasson KH Hilberg O Løkke A Vuust P . Use of Singing for Lung Health as an alternative training modality within pulmonary rehabilitation for COPD: a randomised controlled trial. Eur Respir J. (2022) 59:2101142. 10.1183/13993003.01142-2021 34625480 PMC9117735

[10] Albright RH Fleischer AE A. Primer on cost-effectiveness analysis. Clin Podiatr Med Surg. (2024) 41:313–21. 10.1016/j.cpm.2023.07.006 38388127

[11] Johnson AT. Harmonicas. IEEE Pulse. (2020) 11:32. 10.1109/MPULS.2020.3022204 33064643

[12] Alexander JL Wagner CL. Is harmonica playing an effective adjunct therapy to pulmonary rehabilitation? Rehabil Nurs. (2012) 37:207–12. 10.1002/rnj.33 22744994

[13] Hart MK Stewardson E Jamil AK Tecson KM Millard MW. Usefulness of harmonica playing to improve outcomes in patients with chronic obstructive pulmonary disease. Proc (Bayl Univ Med Cent). (2020) 33:178–82. 10.1080/08998280.2019.1704135 32313456 PMC7155980

[14] Okamoto J Furukawa Y Kobinata N Yoshikawa H Araki F Yagyu A . Combined effect of pulmonary rehabilitation and music therapy in patients with chronic obstructive pulmonary disease. J Phys Ther Sci. (2021) 33:779–83. 10.1589/jpts.33.779 34658524 PMC8516601

[15] Li J Li X Deng M Liang X Wei H Wu X. Features and predictive value of 6-min walk test outcomes in interstitial lung disease: an observation study using wearable monitors. BMJ Open. (2022) 12:e055077. 10.1136/bmjopen-2021-055077 35705338 PMC9204441

[16] Donohue JF. Minimal clinically important differences in COPD lung function. COPD. (2005) 2:111–24. 10.1081/copd-200053377 17136971

[17] Alghamdi SM Janaudis-Ferreira T Alhasani R Ahmed S. Acceptance, adherence and dropout rates of individuals with COPD approached in telehealth interventions: a protocol for systematic review and meta-analysis. BMJ Open. (2019) 9:e026794. 10.1136/bmjopen-2018-026794 31028042 PMC6501945

[18] Pulmonary Function Group of the Chinese Medical Association for Respiratory Diseases. Guidelines for the examination of lung function (second part) – pulmometer examination. Chin J Tuberculosis Respir Dis. (2014) 37:481–6. 10.3760/cma.j.issn.1001-0939.2014.07.00130704229

[19] Pellegrino R Viegi G Brusasco V Crapo RO Burgos F Casaburi R . Interpretative strategies for lung function tests. Eur Respir J. (2005) 26:948–68. 10.1183/09031936.05.00035205 16264058

[20] Zhu Y Zhang Z Du Z Zhai F. Mind-body exercise for patients with stable COPD on lung function and exercise capacity: a systematic review and meta-analysis of RCTs. Sci Rep. (2024) 14:18300. 10.1038/s41598-024-69394-4 39112599 PMC11306772

[21] Enright PL Sherrill DL. Reference equations for the six-minute walk in healthy adults. Am J Respir Crit Care Med. (1998) 158:1384–7. 10.1164/ajrccm.158.5.9710086 9817683

[22] Yang CC Chen HT Luo KH Watanabe K Chuang HY Wu CW . The validation of Chinese version of workplace PERMA-profiler and the association between workplace well-being and fatigue. BMC Public Health. (2024) 24:720. 10.1186/s12889-024-18194-6 38448843 PMC10916278

[23] Vercoulen JH Swanink CM Fennis JF Galama JM van der Meer JW Bleijenberg G. Dimensional assessment of chronic fatigue syndrome. J Psychosom Res. (1994) 38:383–92. 10.1016/0022-3999(94)90099-x 7965927

[24] Cai T Chen J Ni F Zhu R Wu F Huang Q . Psychometric properties of the Chinese version of the functional assessment of chronic illness therapy-fatigue (FACIT-F) among patients with breast cancer. Health Qual Life Outcomes. (2023) 21:91. 10.1186/s12955-023-02164-4 37582752 PMC10428540

[25] Siu DCH So CT Lau CWL Hui EHM Fung A Chan TM . The Manchester respiratory activities of daily living questionnaire: reliability and validity of the Chinese version with pictorial enhancement. Int J Chron Obstruct Pulmon Dis. (2021) 16:91–100. 10.2147/COPD.S283769 33488072 PMC7816045

[26] Liu M Li Y Yin D Wang Y Fu T Zhu Z . COPD assessment test as a screening tool for anxiety and depression in stable COPD patients: a feasibility study. COPD. (2023) 20:144–52. 10.1080/15412555.2023.2174843 37036434

[27] Lin WC Huang TY Liu CY Yeh ML Yu CH Hwang SL. Validation of the clinical COPD questionnaire in Taiwan. COPD. (2016) 13:360–6. 10.3109/15412555.2015.1094456 26678264

[28] van der Molen T Willemse BW Schokker S. ten Hacken NH, Postma DS, Juniper EF. Development, validity and responsiveness of the clinical COPD questionnaire health. Qual Life Outcomes. (2003) 1:13. 10.1186/1477-7525-1-13 12773199 PMC156640

[29] Yang Z Huang X Liu X Hou J Wu W Song A . Psychometric properties and factor structure of the Chinese version of the hospital anxiety and depression scale in people living with HIV. Front Psychiatry. (2019) 10:346. 10.3389/fpsyt.2019.00346 31156484 PMC6531499

[30] Snaith RP. The hospital anxiety and depression scale. Health Qual Life Outcomes. (2003) 1:29. 10.1186/1477-7525-1-29 12914662 PMC183845

[31] Wang X Liu Y Liu Y Zhang J Liu L Matarese M . Exploring patients with COPD self-care behaviours and self-efficacy and their interconnections: a network analysis. J Clin Nurs. (2024) 23:17378. 10.1111/jocn.17378 39041386

[32] Wigal JK Creer TL Kotses H. The COPD self-efficacy scale. Chest. (1991) 99:1193–6. 10.1378/chest.99.5.1193 2019177

[33] Meguro M Barley EA Spencer S Jones PW. Development and validation of an improved, COPD-specific version of the St. George respiratory questionnaire. Chest. (2007) 132:456–63. 10.1378/chest.06-0702 17646240

[34] Xu W Collet JP Shapiro S Lin Y Yang T Wang C . Validation and clinical interpretation of the St George's Respiratory Questionnaire among COPD patients, China. Int J Tuberc Lung Dis. (2009) 13:181−9. 19146745

[35] Yang X Xue M Pauen S He H. Psychometric properties of the Chinese version of multidimensional scale of perceived social support. Psychol Res Behav Manag. (2024) 17:2233–41. 10.2147/PRBM.S463245 38835653 PMC11149633

[36] Zimet GD Powell SS Farley GK Werkman S Berkoff KA. Psychometric characteristics of the multidimensional scale of perceived social support. J Pers Assess. (1990) 55:610–7. 10.1080/00223891.1990.9674095 2280326

[37] Koo TK Li MY A. Guideline of selecting and reporting intraclass correlation coefficients for reliability research. J Chiropr Med. (2016) 15:155–63. 10.1016/j.jcm.2016.02.012 27330520 PMC4913118

[38] Tripepi G Chesnaye NC Dekker FW Zoccali C Jager KJ. Intention to treat and per protocol analysis in clinical trials. Nephrology (Carlton). (2020) 25:513–7. 10.1111/nep.13709 32147926

[39] Yu Z Guindani M Grieco SF Chen L Holmes TC Xu X . Beyond t test and ANOVA: applications of mixed-effects models for more rigorous statistical analysis in neuroscience research. Neuron. (2022) 110:21–35. 10.1016/j.neuron.2021.10.030 34784504 PMC8763600

[40] Rogliani P Laitano R Ora J Beasley R Calzetta L. Strength of association between comorbidities and asthma: a meta-analysis. Eur Respir Rev. (2023) 32:220202. 10.1183/16000617.0202-2022 36889783 PMC10032614

[41] Frei A Radtke T Dalla Lana K Brun P Sigrist T Spielmanns M . Effectiveness of a long-term home-based exercise training program in patients with COPD after pulmonary rehabilitation: a multicenter randomized controlled trial. Chest. (2022) 162:1277–86. 10.1016/j.chest.2022.07.026 35952766

[42] Hariton E Locascio JJ. Randomised controlled trials-the gold standard for effectiveness research: Study design: randomised controlled trials. BJOG. (2018) 125:1716. 10.1111/1471-0528.15199 29916205 PMC6235704

[43] Juul S Gluud C Simonsen S Frandsen FW Kirsch I Jakobsen JC. Blinding in randomised clinical trials of psychological interventions: a retrospective study of published trial reports. BMJ Evid Based Med. (2021) 26:109. 10.1136/bmjebm-2020-111407 32998993

[44] Eze ND Mateus C Cravo Oliveira Hashiguchi T. Telemedicine in the OECD: An umbrella review of clinical and cost-effectiveness, patient experience and implementation. PLoS One. (2020) 15:e0237585. 10.1371/journal.pone.0237585 32790752 PMC7425977

[45] Vallier JM Simon C Bronstein A Dumont M Jobic A Paleiron N . Randomized controlled trial of home-based vs. hospital-based pulmonary rehabilitation in post COVID-19 patients. Eur J Phys Rehabil Med. (2023) 59:103–10. 10.23736/S1973-9087.22.07702-4 36700245 PMC10035444

[46] Arensman RM Geelen RH Koppenaal T Veenhof C Pisters MF. Measuring exercise adherence in patients with low back pain: development, validity, and reliability of the EXercise Adherence Scale (EXAS). Physiother Theory Pract. (2022) 38:928–37. 10.1080/09593985.2020.1818337 32933359

[47] Silva CMDSE Gomes Neto M Saquetto MB Conceição CSD Souza-Machado A. Effects of upper limb resistance exercise on aerobic capacity, muscle strength, and quality of life in COPD patients: a randomized controlled trial. Clin Rehabil. (2018) 32:1636–44. 10.1177/0269215518787338 30012033

[48] Chang J Mai Y Zhang D Yang X Li A Yan W . Media use behavior mediates the association between family health and intention to use mobile health devices among older adults: cross-sectional study. J Med Internet Res. (2024) 26:e50012. 10.2196/50012 38373031 PMC10912999

[49] Wang Y Wu Y Chu H Xu Z Sun X Fang H. Association between health-related quality of life and access to chronic disease management by primary care facilities in mainland china: a cross-sectional study. Int J Environ Res Public Health. (2023) 20:4288. 10.3390/ijerph20054288 36901304 PMC10001723

